# Mechanical allodynia in mice with tenascin-X deficiency associated with Ehlers-Danlos syndrome

**DOI:** 10.1038/s41598-020-63499-2

**Published:** 2020-04-16

**Authors:** Emiko Okuda-Ashitaka, Yuka Kakuchi, Hiroaki Kakumoto, Shota Yamanishi, Hiroki Kamada, Takafumi Yoshidu, Satoshi Matsukawa, Naoya Ogura, Sadahito Uto, Toshiaki Minami, Seiji Ito, Ken-ichi Matsumoto

**Affiliations:** 1 Department of Biomedical Engineering, Osaka Institute of Technology, Osaka, 535-8585 Japan; 2 Department of Anesthesiology, Osaka Medical College, Takatsuki, 569-8686 Japan; 3 Department of Biosignaling and Radioisotope Experiment, Interdisciplinary Center for Science Research, Organization for Research and Academic Information, Shimane University, Izumo, 693-8501 Japan

**Keywords:** Neuroscience, Molecular medicine

## Abstract

Tenascin-X (TNX) is a member of the extracellular matrix glycoprotein tenascin family, and TNX deficiency leads to Ehlers-Danlos syndrome, a heritable human disorder characterized mostly by skin hyperextensibility, joint hypermobility, and easy bruising. TNX-deficient patients complain of chronic joint pain, myalgia, paresthesia, and axonal polyneuropathy. However, the molecular mechanisms by which TNX deficiency complicates pain are unknown. Here, we examined the nociceptive behavioral responses of TNX-deficient mice. Compared with wild-type mice, TNX-deficient mice exhibited mechanical allodynia but not thermal hyperalgesia. TNX deficiency also increased pain sensitivity to chemical stimuli and aggravated early inflammatory pain elicited by formalin. TNX-deficient mice were significantly hypersensitive to transcutaneous sine wave stimuli at frequencies of 250 Hz (Aδ fiber responses) and 2000 Hz (Aβ fiber responses), but not to stimuli at frequency of 5 Hz (C fiber responses). In addition, the phosphorylation levels of extracellular signal-related kinase, an active neuronal marker, and the activity of NADPH-diaphorase, a neuronal nitric oxide activation marker, were enhanced in the spinal dorsal horns of TNX-deficient mice. These results suggest that TNX deficiency contributes to the development of mechanical allodynia and hypersensitivity to chemical stimuli, and it induces hypersensitization of myelinated A fibers and activation of the spinal dorsal horn.

## Introduction

Tenascin-X (TNX) is a member of the extracellular matrix glycoprotein tenascin family, which consists of tenascin-C, tenascin-R, and tenascin-W, and TNX in vertebrates. TNX binds to collagen fibrils and it contributes to architectural functions and tissue integrity in connective tissues within joints, tendons, and skin^1^. TNX deficiency causes an autosomal recessive form of Ehlers-Danlos syndrome (EDS), which is a heritable human connective tissue disorder^2–5^. EDS compasses a clinically and genetically heterogeneous group of diseases, and TNX has been classified as being associated with classical-like EDS through identification of the genetic contributions to symptoms^6^. Classical-like EDS is characterized mostly by skin hyperextensibility, generalized joint hypermobility, and easy bruising without atrophic scarring. Similar to individuals with EDS, TNX-deficient (TNX^−/−^) mice also exhibit progressive skin hyperextensibility due to increased deformability and decreased tensile strength and collagen fibril density^7,8^.

Pain is a common and severe symptom of various types of EDS^9–11^. The pain often initially manifests itself as acute and localized nociception in relation to joint, limb, and soft-tissue trauma, such as subluxations, dislocations, and soft-tissue injury. During the progression of EDS, the pain gradually becomes more persistent and assumes a more generalized distribution^9,10^. It has been reported that EDS patients complain many different manifestations of the pain, such as generalized body pain, soft-tissue pain, dislocation, joint pain, fatigue, neuropathic pain, proprioception loss, headache, and gastrointestinal pain^9^. Chronic pain occurs in approximately 90% of patients with various types of EDS^11^. Classical- or hypermobility-type EDS has been reported to be associated with certain chronic pain conditions such as complex regional pain syndrome (also known as reflex sympathetic dystrophy or causalgia)^12^, fibromyalgia^13,14^, and rheumatic disease^14,15^. TNX-deficient EDS patients complain of chronic joint pain and chronic myalgia^3–5^. Furthermore, neurological features such as peripheral paresthesia and axonal polyneuropathy are frequently observed in the context of TNX-deficient EDS^3,5^. However, the underlying molecular mechanisms of the pain and neurological features of EDS remain poorly understood.

In the present study, we first analyzed pain behavior by using TNX^−/−^ mice to elucidate the contribution of TNX deficiency to the pain associated with EDS. Compared with wild-type mice, TNX^−/−^ mice showed increased pain sensitivity to mechanical and chemical stimuli. We then determined the involvement of TNX in sensory afferent fiber responses to a sine wave electric stimulator and found that TNX^−/−^ mice exhibited hypersensitivity of myelinated Aδ and Aβ fibers. Furthermore, TNX deficiency increased neuronal activation in the spinal dorsal horn of mice. Thus, TNX deficiency induced hypersensitization of myelinated sensory A fibers and central sensitization of the spinal dorsal horn, which induced mechanical allodynia and chemical hyperalgesia.

## Results

### Expression of TNX in the pain transmission pathway

**Figure 1:**
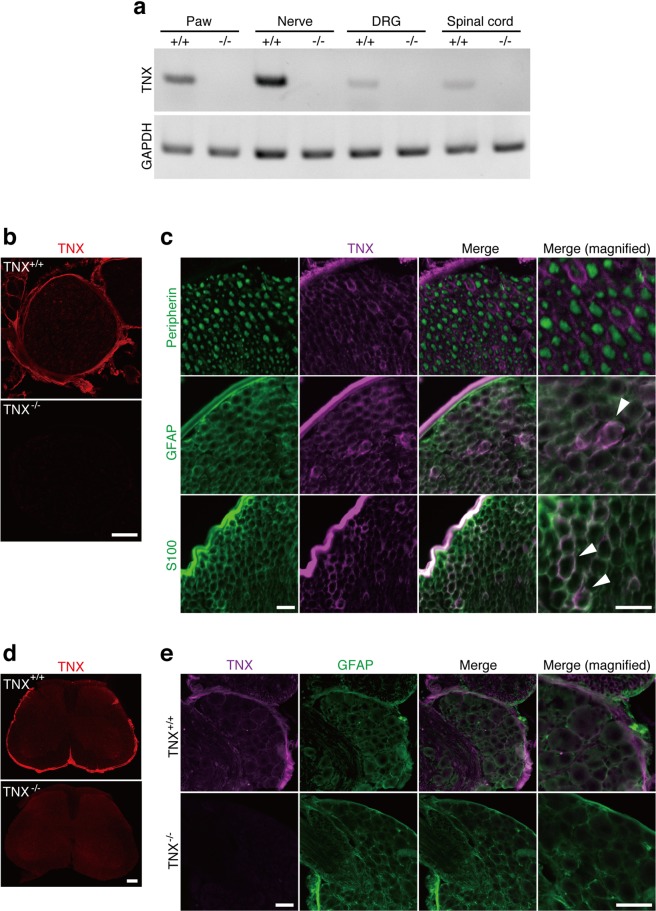
(**a**) Distribution of TNX mRNA. RT-PCR analysis of TNX mRNA expression in the paw, sciatic nerve, DRG, and spinal cord. GAPDH served as the control. Full-length gels are presented in Supplementary Fig. S1. (**b**–**e**) Localization of TNX in the sciatic nerve (**b**,**c**), spinal cord (**d**), and DRG (**e**). Immunofluorescence staining of TNX (red) in the sciatic nerve (**b**) and spinal cord (**d**) of wild-type (TNX^+/+^) and TNX^−/−^ mice. (**c**) Double labeling of TNX (magenta) with peripherin, GFAP, or S100 (green) in sciatic nerve of TNX^+/+^ mice. The arrows indicate the co-expression of TNX and GFAP or S100. (**e**) Double labeling of TNX (magenta) and GFAP (green) in DRG of TNX^+/+^ and TNX^−/−^ mice. Immunohistochemistry was carried out using wild-type and TNX^−/−^ mice (**b** and **d**: n = 4, **c** and **e**: n = 3), and similar results were obtained in each mouse. Scale bar: 100 μm, **b** and **d**; 20 μm, **c** and 50 μm, **e**.

To confirm the expression of TNX mRNA in the pain transmission pathway from peripheral tissues to the spinal cord, we examined the expression of TNX mRNA in the mouse paw, sciatic nerve, dorsal root ganglion (DRG), and spinal cord by using reverse transcription-polymerase chain reaction (RT-PCR) analysis. TNX mRNA was highly detected in the paws and sciatic nerve of wild-type mice (Fig. 1a). In contrast, TNX mRNA was only slightly expressed in the DRG and spinal cord of wild-type mice. TNX mRNAs were not detected in these tissues in TNX^−/−^ mice. We next immunohistochemically examined the localization of TNX in the sciatic nerve, spinal cord, and DRG using an anti-TNX antibody. In the sciatic nerve, TNX was localized in the perineurium and endoneurium of wild-type mice, whereas it was not detected in that of TNX^−/−^ mice (Fig. 1b). To clarify which cells express TNX in the sciatic nerve, we immunohistochemically examined its expression with anti-S100, anti-glial fibrillary acidic protein (GFAP), and anti-peripherin antibodies, markers of myelinating Schwann cells, non-myelinating Schwann cells, and unmyelinated axons, respectively. As shown in Fig. 1c, TNX-immunoreactivity was co-expressed in S100-positive myelinating Schwann cells, a typical round form around axons in the sciatic nerve of wild-type mice. TNX was also co-expressed in GFAP-positive non-myelinating Schwann cells, a discontinuous rings around axons. In contrast, TNX was expressed adjacent to axons expressing peripherin in the sciatic nerve of wild-type mice. These results indicated that TNX was expressed in Schwann cells, but not axons, of peripheral nerve. In the spinal cord of wild-type mice, TNX immunofluorescence was mainly detected in the leptomeninges but not in the white or gray matter (Fig. 1d). TNX immunofluorescence was also detected in the pia matter of DRG and was colocalized with GFAP in the pial fibers (Fig. 1e). TNX immunofluorescence was only faintly detected in satellite cells around the cell bodies of primary sensory neurons, but not in neurons themselves, in the DRG. TNX immunofluorescence was not detected in the sciatic nerve, spinal cord or DRG of TNX^−/−^ mice (Fig. 1b,d and e).

### Physiological pain in TNX^−/−^ mice

**Figure 2:**
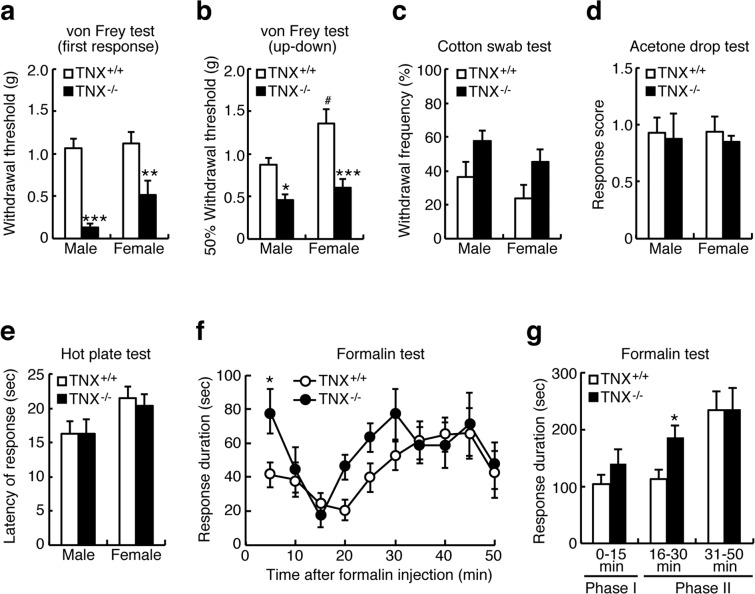
Effects of TNX deficiency on pain responses. (**a**,**b**) The paw withdrawal thresholds of TNX^+/+^ and TNX^−/−^ mice were assessed by the first response method (**a**, male n = 13–14, female n = 10–12), and the 50% withdrawal thresholds were assessed by the up-down method (**b**, male n = 9–11, female n = 10–12) upon mechanical stimulation using von Frey filaments. (**c**) Percent response to a swipe of a puffed-out cotton swab on the hind paw of TNX^+/+^ and TNX^−/−^ mice (male n = 9–11, female n = 10–12). (**d**) Response scores of TNX^+/+^ and TNX^−/−^ mice in the acetone drop test (male n = 5–6, female n = 5–6). (**e**) Response latencies of TNX^+/+^ and TNX^−/−^ mice on a hot plate at 55 °C (male n = 12–13, female n = 10). (**f**,**g**) Pain sensitization induced in TNX^−/−^ mice by peripheral injection of formalin. (**f**) The time spent licking and biting per 5 min after peripheral injection of 2% formalin into the right hindpaw is plotted versus time for TNX^+/+^ and TNX^−/−^ mice (male n = 12). (**g**) Cumulative time spent licking and biting during phase I (0–15 min), early phase II (16–30 min), and late phase II (31–50 min). The data are expressed as the mean ± S.E.M. ***p < 0.001, **p < 0.01, and *p < 0.05, versus the TNX^+/+^ value, ^*#*^*p* < 0.05, versus the male TNX^+/+^ value.

To address whether TNX deficiency affects the behavioral characteristics of physiological pain, we performed a series of behavioral tests on TNX^−/−^ mice, examining sensory responses to a range of mechanical and thermal stimuli. We performed von Frey filament tests to assess the response to mechanical stimulation using two methods: the first response method and the up-down method. In comparison to wild-type mice, TNX^−/−^ male mice showed significantly lower thresholds of nociceptive responses, as assessed by either the lowest force required to elicit a withdrawal reflex (the first response method; wild-type male: 1.07 ± 0.10 g (n = 14) versus TNX^−/−^ male: 0.14 ± 0.04 g (n = 13), *P* < 0.001, one-way ANOVA with *post hoc* Bonferroni test F_(3,45)_ = 15.15, *P* < 0.001, Fig. 2a) or the 50% withdrawal threshold (the up-down method; wild-type male: 0.87 ± 0.09 g (n = 11) versus TNX^−/−^ male: 0.46 ± 0.07 g (n = 9), *P* < 0.05, one-way ANOVA with *post hoc* Bonferroni test F_(3,38)_ = 11.94, *P* < 0.0001, Fig. 2b). As for female mice, TNX^−/−^ female mice also showed significant lower paw withdrawal thresholds (the first response method; wild-type female: 1.12 ± 0.14 g (n = 10) versus TNX^−/−^ female: 0.51 ± 0.18 g (n = 12), *P* < 0.01, Fig. 2a) or the 50% withdrawal threshold (the up-down method; wild-type female: 1.36 ± 0.17 g (n = 10) versus TNX^−/−^ female: 0.60 ± 0.10 g (n = 12), *P* < 0.001, Fig. 2b) in comparison to wild-type female mice. Wild-type female mice were significantly higher paw withdrawal threshold compared than wild-type male mice in the up-down method of von Frey filament test (*P* < 0.05, Fig. 2b). We further examined the responses to innocuous light tactile stimuli by using a puffed-out cotton swab to stroke the paws of mice. As shown in Fig. 2c, both male TNX^−/−^ mice and female ones exhibited a slightly but not significantly greater paw withdrawal frequency than that of wild-type mice (wild-type male: 36.4 ± 8.9% (n = 11) versus male TNX^−/−^: 57.8 ± 6.2% (n = 9), *P* = 0.266; wild-type female: 24 ± 7.8% (n = 10) versus TNX^−/−^ female: 45.0 ± 7.4% (n = 12), *P* = 0.237, one-way ANOVA with *post hoc* Bonferroni test F_(3,38)_ = 3.06, *P* = 0.040). To assess pain sensitivity in response to thermal stimulation, we used an innocuous cold acetone drop test and a noxious heat hot plate test. There was no difference in the response scores to evaporative cooling evoked by acetone application (wild-type male: 0.93 ± 0.14 (n = 6) versus TNX^−/−^ male: 0.88 ± 0.22 (n = 5); wild-type female: 0.94 ± 0.13 (n = 6) versus TNX^−/−^ female: 0.85 ± 0.06 (n = 5), one-way ANOVA with *post hoc* Bonferroni test F_(3,18)_ = 0.088, *P* = 0.966, Fig. 2d) or in the latency to withdrawal from noxious heat at 55 °C (wild-type male: 16.30 ± 1.72 sec (n = 13) versus TNX^−/−^ male: 16.24 ± 2.11 sec (n = 12); wild-type female: 21.44 ± 1.79 sec (n = 10) versus TNX^−/−^ female: 20.32 ± 1.78 sec (n = 10), one-way ANOVA with *post hoc* Bonferroni test F_(3,41)_ = 2.03, *P* = 0.125, Fig. 2e) between wild-type and TNX^−/−^ mice. Collectively, TNX-deficient mice exhibited increased sensitivity to innocuous mechanical stimuli but not to thermal stimuli, suggesting that TNX deficiency induced mechanical allodynia.

### Formalin-induced pain in TNX^/−^ mice

We next subjected wild-type and TNX^−/−^ male mice to a formalin test to determine the involvement of TNX in acute pain induced by peripheral chemical stimuli and persistent inflammatory pain. Injection of formalin (2%, *s.c*.) into the dorsal surface of the hindpaw evoked nociceptive behaviors, namely licking and biting of the injected paw, in a biphasic manner. The formalin response consisted of an initial transient phase (phase I) during the first 10 min followed by a prolonged phase (phase II) beginning 15 min after injection of formalin (Fig. 2f). Both wild-type and TNX^−/−^ mice exhibited biphasic pain responses in the formalin test. As shown in Fig. 2f, the duration of pain behavior during the phase I, in particular during the first 5 min, was significantly greater in TNX^−/−^ mice than that in wild-type mice (wild-type: 41.2 ± 7.3 s (n = 12) versus TNX^−/−^: 78.7 ± 12.9 s (n = 12), *P* < 0.05, two-way ANOVA with *post hoc* Bonferroni test F_(9, 99)_ = 3.63, *P* = 0.0006). In phase II, the duration of pain behavior at the early stage (16–30 min), but not at the late stage (31–50 min), was significantly increased in TNX^−/−^ mice compared with that in wild-type mice (wild-type: 112.7 ± 16.8 s versus TNX^−/−^: 186.1 ± 21.2 s, *P* < 0.05, one-way ANOVA with *post hoc* Bonferroni test F_(5,66)_ = 4.92, *P* = 0.0007, Fig. 2g). Phase I of the formalin response is linked to direct activation of primary afferent sensory neurons, while phase II is associated with inflammatory responses mediated by the combined effects of primary afferent inputs and central sensitization in the spinal dorsal horn^16^. Taken together, these findings indicate that TNX deficiency increased pain sensitivity to acute stimulation of nociceptors and aggravated the early inflammatory pain elicited by formalin.

### Effects of analgesic agents on mechanical allodynia in TNX^−/−^ mice

**Figure 3:**
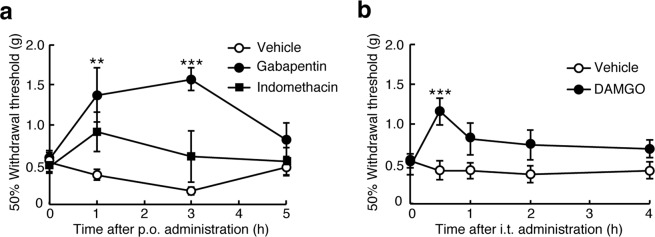
Effects of analgesic agents on mechanical allodynia in TNX^−/−^ mice. (**a**) Gabapentin (50 mg/kg), indomethacin (5 mg/kg), or vehicle was orally (*p.o*.) administrated in TNX^−/−^ mice (n = 5). (**b**) DAMGO (1 μg) or vehicle was intrathecally (*i.t*.) administrated in TNX^−/−^ mice (n = 8). 50% paw withdrawal thresholds were measured at the indicated times using von Frey test (up-down methods). The data are expressed as the mean ± S.E.M. **p < 0.01, ***p < 0.001 versus the saline-administrated value.

To further clarify the pharmacological profile of mechanical allodynia in TNX^−/−^ mice, we performed the pharmacological experiments with an anticonvulsant drug gabapentin, a non-steroidal anti-inflammatory drug indomethacin, and a Mu-opioid receptor agonist DAMGO ([D-Ala^2^, N-Me-Phe^4^, Gly-ol^5^]enkephalin. Gabapentin binds to α_2_δ-1 subunit of voltage-dependent calcium channel, which inhibits neuropathic pain^17^. As shown in Fig. 3a, oral administration of gabapentin (50 mg/kg) markedly inhibited mechanical allodynia, compared with that of vehicle, in TNX^−/−^ mice, which was significant at 1 h and 3 h after the administration (1 h, vehicle: 0.37 ± 0.07 g (n = 5) versus gabapentin: 1.37 ± 0.34 g (n = 5), *P* < 0.01; 3 h, vehicle: 0.17 ± 0.05 g versus gabapentin: 1.57 ± 0.14 g, *P* < 0.001, two-way ANOVA with *post hoc* Bonferroni test F_(6,36)_ = 3.26, *P* = 0.012). In contrast, oral administration of indomethacin (5 mg/kg) did not significantly affect mechanical allodynia in TNX^−/−^ mice (n = 5), whereas the same concentration of indomethacin blocked the allodynia induced by human immunodeficiency virus type-1 glycoprotein gp 120^18^. Furthermore, intrathecal injection of DAMGO (1 μg) inhibited mechanical allodynia compared with that of vehicle in TNX^−/−^ mice, which was significant at 30 min after administration (vehicle: 0.42 ± 0.12 g (n = 8) versus DAMGO: 1.16 ± 0.17 g (n = 8), *P* < 0.001, two-way repeated ANOVA with *post hoc* Bonferroni test F_(4,56)_ = 3.13, *P* = 0.022, Fig. 3b). These results indicate that the TNX deficiency-induced mechanical allodynia is sensitive to gabapentin and the Mu-opioid agonist DAMGO, but not indomethacin.

### **Hypersensitization of A-fiber in TNX**^−/−^**mice**

**Figure 4:**
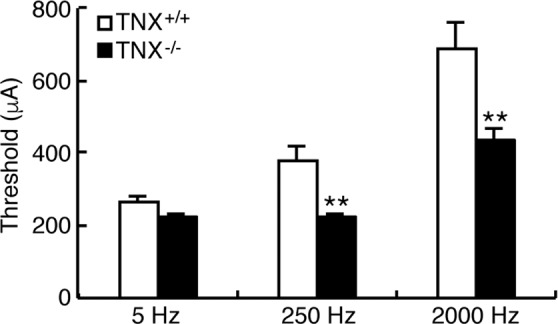
Paw withdrawal thresholds in response to sine-wave electrical stimuli in TNX^−/−^ mice. The current threshold represents the minimum intensity (μA) required to produce a paw withdrawal response with sine wave electrical stimulation at 5, 250, and 2000 Hz. The data are expressed as the mean ± S.E.M. (n = 6). **p < 0.01 versus the TNX^+/+^ value.

The pain behavior assays revealed differences in sensitivity between TNX-deficient and wild-type mice, as shown in Fig. 2; in particular, TNX-deficient mice exhibited increased sensitivity to innocuous mechanical stimuli but not to noxious thermal stimuli. The perception of acute noxious heat stimuli is typically initiated by unmyelinated C fibers and thinly myelinated Aδ fibers, and in turn induces nociceptive responses^19,20^. Sensing of innocuous tactile stimuli is mainly conducted through myelinated Aβ fiber, which have fast conduction velocities and low activation thresholds. Therefore, we examined the responses of sensory afferent fibers using transcutaneous sine wave stimuli. Transcutaneous nerve stimulation was conducted with three sine wave pulses with frequencies of 5, 250, 2000 Hz to activate C, Aδ, and Aβ fibers, respectively^21,22^. The three different frequencies were applied to the left hindpaws of the mice, and the intensity was gradually increased. As shown in Fig. 4, the application of 5-Hz stimulus to the hindpaw of TNX^−/−^ mice yielded a slight but insignificant decrease in the paw withdrawal threshold compared to that of wild-type mice (wild-type: 266.2 ± 17.8 μA versus TNX^−/−^: 223.0 ± 10.2 μA, *P* = 0.850, one-way ANOVA with *post hoc* Bonferroni test F_(5.30)_ = 18.66, *P* < 0.0001). In contrast, the thresholds for the 250- and 2000-Hz stimulus responses were significantly reduced in TNX^−/−^ mice compared with those in wild-type mice (250 Hz, wild-type: 379.3 ± 40.1 μA versus TNX^−/−^: 225.6 ± 9.8 μA, *P* < 0.01; 2000-Hz, wild-type: 691.8 ± 54.78 μA versus TNX^−/−^: 433.0 ± 33.3 μA, *P* < 0.01, one-way ANOVA with *post hoc* Bonferroni test F_(5.30)_ = 18.66, *P* < 0.0001). Thus, TNX-deficient mice mainly exhibited hypersensitization of myelinated Aδ and Aβ fibers, but not unmyelinated C fibers.

### **Central sensitization in the spinal dorsal horns of TNX**^−/−^**mice**

**Figure 5:**
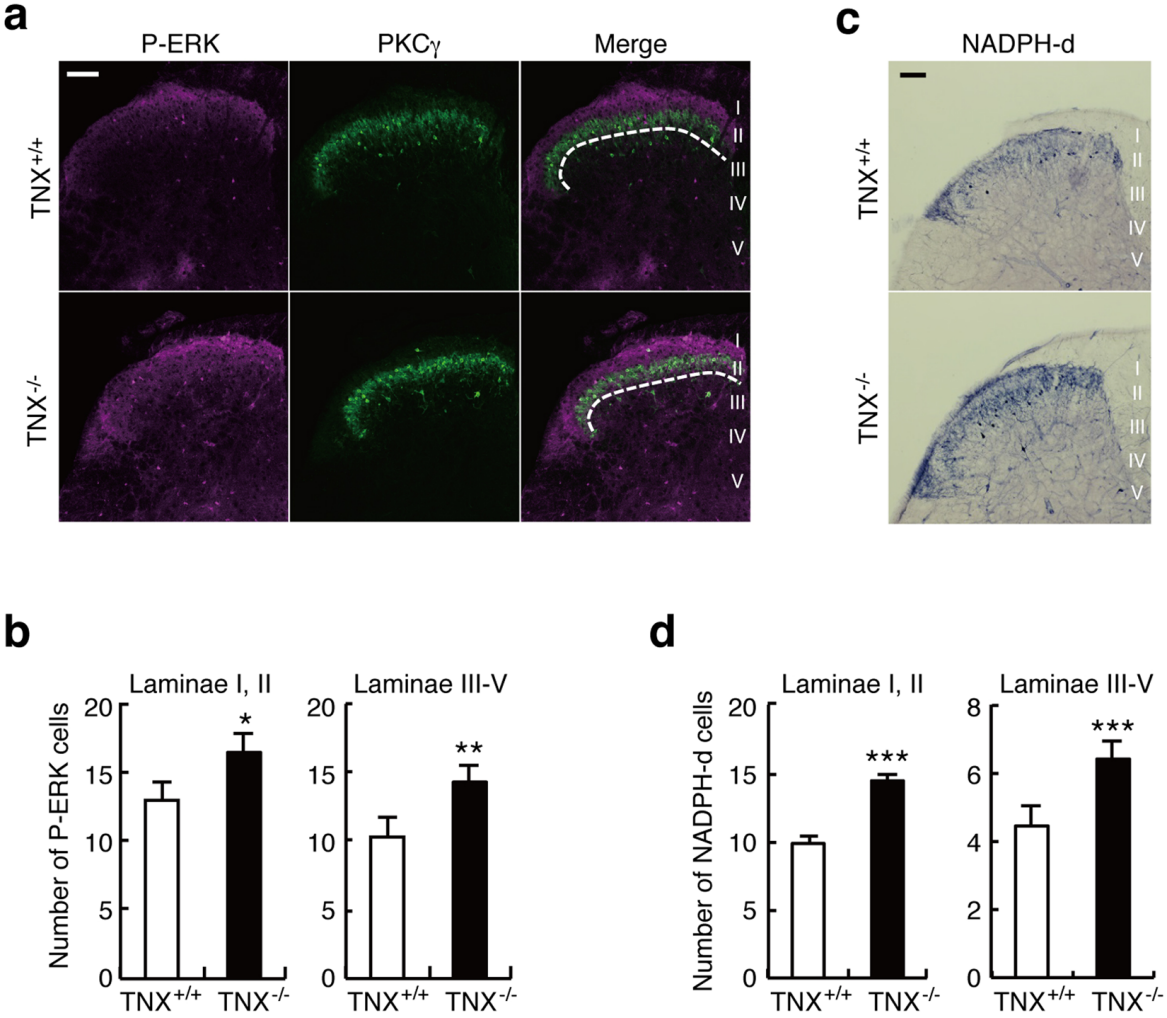
Central sensitization in the spinal dorsal horns of TNX^−/−^ mice. (**a**,**b**) ERK phosphorylation in the spinal dorsal horns of TNX^+/+^ and TNX^−/−^ mice. (**a**) Representative fluorescence images of p-ERK (magenta) and PKCγ (green) immunoreactivity in the dorsal horn of the spinal cord. PKCγ was used as a marker of lamina IIi. Dotted lines show the border between lamina II and lamina III. (**b**) The numbers of p-ERK-positive neurons in laminae I, II and III-V of the spinal dorsal horn. p-ERK immunoreactivity was quantified in 30 images of 15 slices prepared from the 3 separated mice. (**c**,**d**) NADPH-diaphorase (NADPH-d) staining, which reflects nNOS activity, in the spinal dorsal horns of TNX^+/+^ and TNX^−/−^ mice. (**c**) Representative fluorescence images of NADPH-diaphorase staining in the dorsal horn of the spinal cord. (**d**) NADPH-diaphorase-positive cells were quantified in 27 (wild-type) or 32 (TNX^−/−^) images of 16 slices prepared from the 4 separated mice. The data are expressed as the mean ± S.E.M. ***p < 0.001, **p < 0.01 and *p < 0.05 versus the TNX^+/+^ value. Scale bar, 100 μm.

The dorsal horn of the spinal cord is an important site for pain transmission, and it constitutes the first relay station for incoming somatosensory information^19,20^. High-threshold C and Aδ fibers project to the superficial dorsal horn laminae I and II, while low-threshold C and Aδ fibers the inner zone of laminae II (IIi) and III. Although Aβ fibers send information directly through the dorsal columns to the brainstem, they project laterally to the deeper dorsal horn laminae from lamina IIi to IV and polysynaptically connect to lamina I projection neurons. We used phosphorylated extracellular signal-regulated kinase (p-ERK), the anatomical neuronal activation marker, to investigate central sensitization in the spinal dorsal horns of TNX-deficient mice. As shown in Fig. 5a,b, compared with wild-type mice, TNX-deficient mice exhibited significantly greater numbers of p-ERK-immunoreactive neurons in laminae I and II and in laminae III-V (laminae I, II, wild-type: 12.9 ± 1.33 (n = 30) versus TNX^−/−^: 16.4 ± 0.96 (n = 30), *t*_58_ = 2.1, *P* < 0.05; laminae III-V: wild-type, 10.3 ± 0.78 versus TNX^−/−^: 14.3 ± 0.93, *t*_58_ = 3.2, *P* < 0.01). Furthermore, we also analyzed neuronal nitric oxide synthase (nNOS) activity in the spinal dorsal horns of TNX^−/−^ mice (Fig. 5c,d). nNOS activation in the superficial dorsal horn of the spinal cord is involved in the maintenance of neuropathic pain after nerve injury, and it is triggered by the increases in intracellular Ca^2+^ concentration through N-methyl-D-aspartate receptors^23^. Paraformaldehyde-resistant NADPH-diaphorase activity is identical to nNOS activity in the central nervous system, therefore nNOS activity was assessed as NADPH-diaphorase activity^24^. As shown in Fig. 5c,d, the numbers of NADPH-diaphorase-positive neurons were evidently increased in the dorsal horns of TNX^−/−^ mice as compared with those of wild-type mice (laminae I and II, wild-type: 9.9 ± 0.58 (n = 27) versus TNX^−/−^: 14.4 ± 0.50 (n = 32), *t*_57_ = 5.7, *P* < 0.001; laminae III-V, wild-type: 4.4 ± 0.30 versus TNX^−/−^: 6.4 ± 0.37, *t*_57_ = 4.0, *P* < 0.001). Thus, TNX-deficient mice increased activations of ERK and nNOS in the dorsal horn from lamina I to lamina V of spinal cord, demonstrating that TNX deficiency induced spinal central sensitization.

## Discussion

In this study, we first analyzed pain-like behaviors using TNX^−/−^ mice, a murine EDS model, and obtained novel evidence that TNX deficiency is involved in mechanical allodynia, chemical stimulus-induced hyperalgesia and inflammatory pain. Compared with wild-type mice, TNX^−/−^ mice showed greater pain sensitivity to innocuous peripheral mechanical stimuli. TNX deficiency also increased pain sensitivity to peripheral chemical stimulation by formalin. In contrast, TNX^−/−^ mice showed normal pain sensitivity to thermal stimuli such as innocuous cold and noxious heat. The TNX deficiency-induced mechanical allodynia was inhibited by the anticonvulsant drug gabapentin and the Mu-opioid agonist DAMGO. Furthermore, TNX deficiency was determined to be involved in hypersensitization of primary sensory neurons and central sensitization of spinal dorsal horn neurons. TNX^−/−^ mice were significantly more sensitive than wild-type mice to transcutaneous sine wave stimuli at frequencies of 250 Hz and 2000 Hz, which elicited myelinated A fiber responses. TNX deficiency also increased ERK and nNOS activation in spinal dorsal horn neurons.

Neurological features such as peripheral paresthesia and axonal polyneuropathy are frequently observed in the context of TNX-deficient EDS^3,5^. Approximately 68% of EDS patients with chronic pain have neuropathic pain, the major symptom of which is mechanical allodynia^9^. TNX^−/−^ mice showed increased pain sensitivity to mechanical stimulation with von Frey filaments under basal conditions (Fig. 2a,b), suggesting that mechanical allodynia similar to that in human EDS patients was evoked in this murine TNX-deficient EDS model. Further, male-female percentage of human TNX-deficient EDS patients are 35% of male and 65% of female, and both genders exhibit chronic pain, peripheral paresthesia, and axonal polyneuropathy^3^. There are no marked gender differences in clinical manifestations of TNX-deficient EDS patients. In murine pain behavior assays (Fig. 2a–e), TNX deficiency showed similar effects of pain responses in both male and female mice compared with respective wild-type mice. The mechanical allodynia was also observed in both male and female TNX^−/−^ mice. Taken together, the murine TNX-deficient EDS model is useful to understand the mechanisms and therapeutic targets of pathological pain associated with EDS.

TNX deficiency-induced mechanical allodynia was inhibited by the anticonvulsant drug gabapentin (50 mg/kg, *p.o.*) and the Mu-opioid agonist DAMGO (1 μg, *i.t.*), but not by the non-steroidal anti-inflammatory drug indomethacin (5 mg/kg, *p.o.*), as shown in Fig. 3. It has been reported that gabapentin and DAMGO are effective in neuropathic pain, in contrast indomethacin is no effective. Oral administration of gabapentin (10–120 mg/kg), but not indomethacin (1–10 mg/kg), reduces mechanical allodynia in spinal nerve ligation model of neuropathic pain^25,26^. Intrathecal administration of DAMGO (1 μg) inhibits mechanical hypersensitivity in the spinal nerve ligation model^27^. These pharmacological results suggest that TNX deficiency induces mechanical allodynia with characteristics of neuropathic pain. In addition to the neuropathic pain, TNX deficiency increased pain sensitivity to chemical stimuli and aggravated the early inflammatory pain elicited by 2% *s.c.* formalin (Fig. 2f,g). Besides theses acute effects of formalin (up to 50 min), 2% formalin has been reported to produce long-lasting mechanical allodynia and hyperalgesia for at least 2 week^26^. The formalin-induced long-lasting secondary hypersensitivity is inhibited by gabapentin, but not by indomethacin. TNX deficiency might affect the formalin-induced neuropathic pain, although this possibility requires further investigation.

Neuropathic pain is related to changes in the current thresholds of sensory fibers. In previous studies, neuropathic pain model mice has been found be to be decreased in the 2000-Hz (Aβ fibers) and 250-Hz (Aδ fibers) current thresholds and increased or unchanged in 5-Hz (C fibers) current thresholds compared with those of control mice^28,29^. In the present study, TNX deficiency significantly decreased the current thresholds for responses to transcutaneous sine wave stimuli at frequencies of 250 Hz and 2000 Hz (Fig. 4), suggesting that TNX deficiency affected myelinated Aβ and Aδ fibers. In contrast, the thresholds for responses to transcutaneous sine wave stimuli at frequencies of 5 Hz were unaffected in TNX^−/−^ mice (Fig. 4). Transcutaneous nerve stimuli with frequencies of 5 Hz activate C fibers^21,22^. In addition, neonatal treatment with capsaicin to eliminate C fibers in mice increases the threshold for responses to 5-Hz stimuli^29^. Furthermore, mice treated neonatally with capsaicin exhibit a prolonged latency period in the hot plate test, indicating that the response to the hot plate is transduced via C fibers^30^. In this study, the latency period to the noxious heat stimulus in the hot plate test was unaffected in TNX^−/−^ mice (Fig. 2e). Thus, TNX-deficiency does not alter the activation of C fibers by noxious thermal stimulation at 5 Hz. On the other hand, oral administration of gabapentin inhibited mechanical allodynia in TNX^−/−^ mice (Fig. 3a). Gabapentin blocks the neuropathic pain induced by a chemotherapeutic agent paclitaxel^31^. Paclitaxel induces hypersensitization of Aβ and Aδ fibers but not C fiber, and the A fibers specific hypersensitization is blocked by gabapentin. Intrathecal injection of DAMGO, a Mu-opioid receptor agonist, inhibited mechanical allodynia in TNX^−/−^ mice (Fig. 3b). DAMGO inhibits presynaptically monosynaptic Aδ- and C-fiber-evoked excitatory postsynaptic current in lamina I and II of spinal cord^32,33^. Taken together, these findings indicate that mice with TNX deficiency may develop mechanical allodynia via hypersensitization of myelinated A fibers.

Sensing of innocuous light touches is mainly conducted through myelinated Aβ fibers, while the perception of acute noxious thermal mechanical, and chemical stimuli is typically initiated by unmyelinated C fibers and thinly myelinated Aδ fibers^19,20^. Administration of formalin (2%, *s.c*.) significantly increased the pain response during phase I, particularly at 1–5 min, in TNX^−/−^ mice (Fig. 2f). It has been reported that high dose of formalin (2.5%), which is more commonly used in the formalin test, activates myelinated Aδ and Aβ fibers as well as unmyelinated C fibers^34^. Nociceptive Aδ and C fibers exhibit sustained firing during both phase I and phase II of the formalin test, and non-nociceptive Aβ fibers  are activated during phase I. Furthermore, formalin interacts directly transient receptor potential ankyrin 1 (TRPA1) which is expressed in subpopulation of C and Aδ fibers^35^. Administration of TRPA1 antagonist and the TRPA1-deficency decreases the formalin-evoked pain behaviors in both phase I and phase II. TNX^−/−^ mice decreased in the 2000-Hz (Aβ fibers) and 250-Hz (Aδ fibers) current thresholds and unchanged in 5-Hz (C fibers) current thresholds (Fig. 4). Taken together, these findings suggest that TNX deficiency increased formalin-induced pain sensitivity via hypersensitization of myelinated A fibers.

TNX was not detected in sensory neurons of the DRG or spinal cord, which contain the somata of primary sensory neurons (Fig. 1d,e). Our results are supported by previous data showing that TNX does not directly colocalize with nociceptive afferent fibers expressing calcitonin gene-related peptides in the mouse distal colon^36^. TNX was expressed in two types of glial cells, satellite glial cells in DRG and Schwann cells in the peripheral nerve (Fig. 1c–e). Sakai *et al*. also has reported that TNX is expressed in Schwann cells in the sciatic nerve^37^. Schwann cells support axonal outgrowth and myelination of the large associated axons, and hence abnormal packing of myelin sheaths has been reported in the cutaneous nerves of the TNX-deficient patients with classical-like EDS^2^. Morphological analysis by using electron microscopy has revealed slight differences between the myelinated fibers of TNX^−/−^ mice and those of wild-type mice. For example, the myelinated sciatic nerve fibers in the TNX-deficient mice exhibit modestly smaller inner and outer diameters than those in wild-type mice^38^, although the numbers of axons and the thickness of the myelin sheaths are not significantly different in TNX^−/−^ mice^39^. In the present study, TNX deficiency decreased the current thresholds in the myelinated A fibers (Fig. 4). Notably, disorder of myelin contributes to the development of neuropathic pain^40^. Myelin disorder is associated with expression changes of pain-related molecules including channels, receptors, cytokines, and growth factors, and ephaptic interaction between innocuous Aβ fibers and noxious Aδ and C fibers^41^. Thus, in summary, dysplasia of myelination may have induced the A fiber hypersensitivity and mechanical allodynia in TNX^−/−^ mice.

TNX deficiency also induced central sensitization in the dorsal horn of the spinal cord. The numbers of p-ERK- and NADPH-d-positive neurons were increased in the dorsal horn in TNX^−/−^ mice under basal conditions (Fig. 5). Nociceptive information is mainly mediated by Aδ and C fibers that terminate in the laminae I and II and activate projection neurons and excitatory interneurons, whereas innocuous mechanical information is mediated by Aβ fibers that terminate in the laminae IIi-IV and polysynaptically connect to lamina I projection neurons via excitatory and inhibitory interneurons^20^. The increases in pERK- and NADPH-d-positive neurons were observed in the dorsal horn from lamina I to lamina V (Fig. 5), suggesting that the central sensitization induced by TNX deficiency encompassed areas of input for primary sensory neurons. Furthermore, in the formalin test, the duration of pain behavior in the early stage of phase II was greater in TNX^−/−^ mice than in wild-type mice (Fig. 2g). Phase II is associated with inflammatory responses mediated by the combined effects of primary afferent input and central sensitization in the spinal dorsal horn^16^. Overall, these findings indicate that the central sensitization was involved in the mechanical allodynia and formalin-induced inflammatory pain in TNX^−/−^ mice.

In addition to sensitivity of A fibers sensitization and central sensitization, other possible mechanisms underlying the enhanced response to chemical stimuli include the expression or sprouting of nociceptive sensory neurons and the regulation of leptomeninges. The pain response during phase I of formalin test, particularly during first 5 min, was increased in TNX^−/−^ mice (Fig. 2f). Phase I of the formalin response involves direct activation and acute sensitization of nociceptors in response to chemical stimuli. It has been recently reported that colonic afferent sensitivity is increased in the TNX^−/−^ mice compared with wild-type mice^36^. Specifically, the density of nociceptive fibers expressing calcitonin gene-related peptides is increased in the colonic mucosa but not in the myenteric plexus. TNX may regulate the sprouting of nociceptive neurons responsive to chemical stimuli; this possibility requires further investigation into sensory neuron expression in peripheral terminal tissues. On the other hand, TNX was predominantly expressed in the leptomeninges of spinal cord and the pia matter of DRG (Fig. 1d,e). Consistent with our results, TNX has been reported to localize in the meninges of the spinal cord^42^, in the leptomeningeal trabeculae of the cerebral cortex and in the connective tissue of the lateral ventricle choroid plexus^43^. The leptomeninges have a high elastic modulus, and they play important roles as barriers and facilitators for the movement of fluid, cells, and pathogens^44^. A relationship between leptomeninges and neuropathic pain has been reported. Selective infiltration of CD4+ αβ T cells into the dorsal root leptomeninges of somatosensory pathways induces the mechanical allodynia after peripheral nerve injury^45^. Taken together, the data suggest that TNX-mediated regulation of the construction and function of the leptomeninges may induce mechanical allodynia, although this possibility requires further investigation.

In conclusion, we determined that TNX contributes to the mechanical allodynia, the formalin-induced hyperalgesia, and the aggravation of inflammatory pain by using the TNX^−/−^ mice. TNX deficiency modulated at least two mechanisms, hypersensitization of myelinated sensory A fibers and spinal central sensitization via ERK activation and nNOS activation. Our findings suggest that TNX is a therapeutic target for the management of pathological pain associated with EDS.

## Methods

### Animals

All experiments were carried out in accordance with the National Institutes of Health Guide for the Care and Use of Laboratory Animals, and were approved by the Animal Experimentation Committee of Osaka Institute of Technology. TNX^−/−^ mice were generated as previously reported^46^. The TNX^−/−^ mice were backcrossed with C57BL/6 J for 10 generations. Age-matched C57BL/6 J mice were used as wild-type (TNX^+/+^) mice. The animals were housed under conditions of a 12-h light/12-h dark cycle and a constant temperature of 22 ± 2 °C. They were allowed free access to food and water before testing. Six to 10-week-old male and female mice were used for the behavioral studies of physiological pain, and male mice were used for other experiments. The animal experiments were conducted in accordance with the ethical guidelines of the Ethics Committee of the International Association for the Study of Pain. The experimenter was blinded to the genotypes of the mice.

### von Frey test

Mice were randomly placed individually in a glass chambers that were placed on a mesh floor. The mice were habituated to the test environment for 30 min. Mechanical nociception was assessed by the first response method^47^ and the up-down method^48^ using von Frey filaments. In the first response method, calibrated von Frey filaments were applied in ascending order through the mesh floor to the plantar surface of the hindpaw 5 times at intervals of a few seconds. The withdrawal threshold was defined, as the lowest force required to elicit a withdrawal reflex of the paw. A lack of response to the 2.0-g filament was assigned a value of 2.0 g. In the up-down method, the mechanical sensitivity was evaluated using calibrated von Frey filaments (0.02–2.0 g). The first stimulus was always the 0.4-g filament. When a withdrawal reflex of the paw was elicited, the next lower-rated filament was applied, and when there was no response, the next higher-rated filament was used. After the first change in the direction of response, an additional 4 measurements were carried out, and the 50% withdrawal threshold was calculated by the up-down method^48^. The experiments using different mice groups were carried out at least three times, and similar results were obtained.

### Cotton swab test

Behavioral responses to light touch were analyzed by using a puffed-out cotton swab as described previously^49^. Mice were placed in a glass chamber on a mesh floor and allowed to acclimatize for 30 min before testing. A cotton swab was expanded to approximately 3 times its original size, and the mice assayed for paw withdrawal response after the swab was stroked for <1 s along the plantar paw surface in a heel to toe direction. The stroking movements were repeated 5 times, alternating between paws, with at least a 1-min interval. The number of paw withdrawal events during the 5 repeated tests was counted and recorded as a percentage (%) for each mouse.

### Hot plate test

Noxious heat nociception was examined with a hot plate test^50^. Mice were placed on top of a hot plate at 55 °C, and the latency of responses, as distinguished by flicking or licking of the hindpaw or jumping, was measured. A cutoff time of 30 s was set in order to prevent tissue injury to the mice. Each animal was measured one time in the hot plate to avoid sensitization to heat response induced by repeat stimulation of high temperature.

### Acetone drop test

Cold nociception was examined with an acetone drop test as described previously^51^. Mice were acclimatized to individual observation cages for 30 min and a drop (50 μl) of acetone was placed against the center of the plantar surface of the hindpaw of each mouse. The responses of the mice were monitored for 40 sec after acetone application. The behavioral responses were scored according to the following scale: 0, no response; 1, quick paw withdrawal or a single flick; 2, repeated flicking of the paw; and 3, licking of the plantar surface of the paw. The test was repeated five times with alternation of the hindpaw and mouse between trials. The mean of response score was then generated for each mouse.

### Formalin test

A formalin test was performed essentially according to the procedure of Nakano *et al*.^52^. Mice were acclimatized to individual observation cages for 30 min and were then subcutaneously (*s.c*.) injected with 20 μl of 2% formalin in 0.9% saline into the dorsal surface of the right hindpaw. The time spent by each mouse on licking and biting behaviors was recorded at 5-min intervals for 50 min. The acute phase (up to approximately 15 min after formalin injection) was defined as phase I. The phase of persistent inflammation, which increased after 16 min and persisted up to 50 min, was defined as phase II.

### Drug administration

Gabapentin and indomethacin were purchased from Fujifilm Wako Pure Chemical Corporation (Osaka, Japan), and DAMGO from Sigma-Aldrich (St. Louis, MO). Gabapentin and DAMGO were dissolved in saline, and indomethacin in 10% ethanol/saline. For oral administration, gabapentin or indomethacin solutions were diluted to 10-fold in 0.5% methylcellulose, and they were given to mice using feeding needles (170 μl/mice; gabapentin (50 mg/kg), indomethacin (5 mg/kg)). DAMGO (5 µl, 1 μg) was intrathecally injected into the subarachnoid space between the L5 and L6 vertebrae using 27-gauge stainless steel needle attached to a microsyringe according to the procedure reported previously^53^.

### Electrical stimulation-induced paw withdrawal test

Mice were placed into a polypropylene chamber and the left paw of each mouse was drawn thorough a hole in the chamber. After 5-min, electrodes (3 mm in diameter) were attached to the left plantar and dorsal surfaces of the hindpaw of each mouse using GEL103 conductive adhesive gel (BIOPAC Systems, Goleta, CA). Transcutaneous nerve stimuli using each of three sine wave currents (5, 250, and 2000 Hz) were applied for 3 s through the electrodes. The sine wave voltage generated by the function generator FG-274 (Texio Technology Corporation, Yokohama, Japan) was converted to the sine wave current using an operational amplifier (Texas Instruments OPA445, Dallas, TX). Regardless of the electrical resistance of the mouse, this system produced a current proportional to the voltage. The current intensity was increased gradually, and the minimum current intensity of the paw withdrawal response was defined as the paw withdrawal threshold. Transcutaneous nerve stimuli using each sine wave currents were applied to the paw at 5-min intervals. The same experiments were repeated four times for the same mice in the experiment day and the next day (twice a day at a 4-h interval). The paw withdrawal threshold was averaged from 4 trials per paw.

### RT-PCR

Total RNAs were extracted from mouse tissues using TRIzol reagent (ThermoFisher Scientific, Waltham, MA), and the first-strand cDNA was synthesized from 1 μg of total RNA by using a ReverTra Ace (Toyobo, Osaka, Japan). First-strand cDNA was amplified in a buffer containing *Gene Taq* DNA polymerase (Nippon Gene, Tokyo, Japan) and an anti-*Taq* antibody (anti-*Taq* high; Toyobo) with primers as follows: 5′-TGGAGGAGCTGGTAAAAGGG-3′ and 5′-CTTCGGGACAGGACTTGGAG-3′ for TNX; 5′-AATGTGTCCGTCGTGGATCTG-3′ and 5′-TGGTCCAGGGTTTCTTACTCC-3′ for glyceraldehyde-3-phosphate dehydrogenase (GAPDH). RT-PCR-amplifications were performed as follows: 1 cycle at 94 °C for 1 min followed by 35 cycles at 94 °C for 1 min and 55 °C for 1.5 min, and at 72 °C for 30 s, and finally 1 cycle at 72 °C for 7 min. PCR products (234 bp for TNX and 308 bp for GAPDH) were separated on 1.5% agarose gels in Tris-acetate-EDTA buffer.

### Immunohistochemistry

Immunohistochemistry was essentially performed as previously described^50^. Briefly, mice were deeply anesthetized using sodium pentobarbital and then intracardially perfused with phosphate-buffered saline (PBS) followed by 4% paraformaldehyde in 0.1 M phosphate buffer (pH 7.4). The lumbar spinal cord and the DRG were dissected, fixed again in 4% paraformaldehyde overnight, and cryoprotected in 30% sucrose overnight. The nerve segments were post-fixed by immersion in the 4% paraformaldehyde paraformaldehyde overnight, and cryoprotected in 30% sucrose overnight. Spinal cord sections (40 μm thick) were prepared using a sliding microtome. DRG and nerve sections (10 µm thick) were prepared using a cryostat, and these sections were mounted on aminopropylsilane-coated glass slides (Matsunami, Osaka, Japan). The free-floating spinal cord or DRG and nerve sections on glass slides were blocked with PBS containing 10% normal goat serum and 0.2% Triton X-100 and then incubated with primary antibodies overnight. The primary antibodies included rabbit anti-TNX (1:250, Matsumoto *et al*.^39^), chicken anti-peripherin (1:250, abcam, Camgrige, UK, ab39374), anti-GFAP (1:500, Millipore, Burlingtonm MA, MAB360), mouse monoclonal anti-S100 (1:500, Sigma-Aldrich, #S2532), rabbit anti-p-ERK (1:1000, Cell Signaling Technology, Danvers, MA, #9101), and guinea pig anti-protein kinase Cγ (PKCγ, 1:200, Frontier Institute, Hokkaido, Japan, #AB2571826). The immune complexes were visualized using Alexa 546-labeled anti-rabbit IgG (1:1000, ThermoFisher Scientific, #A11035), Alexa 488-labeled anti-mouse IgG (1:1000, ThermoFisher Scientific, #A11029), Alexa 488-labeled anti-guinea pig IgG (1:800, Jackson ImmunoResearch Laboratories, West Grove, PA, #706–545–148), and Alexa 488-labeled anti-chicken IgY (1:1000, ThermoFisher Scientific, #A11039) secondary antibodies. Digital images were captured using a Nikon A1 laser-scanning confocal microscope equipped with an argon HeNe1 laser and an appropriate filter (Nikon Corporation, Tokyo, Japan).

### NADPH-diaphorase histochemistry

Neuronal nitric oxide synthase activity and distribution in the spinal cord were determined by using NADPH-diaphorase histochemistry, as previously described^54^. Tissue sections (40 µm thick) were cut on a sliding microtome and were incubated in a solution containing 0.5 mg/mL β-NADPH, 0.2 mg/mL nitroblue tetrazolium and 0.25% Triton X-100 in PBS for 90 min at 37 °C. Images were captured with a color digital camera on mounted a microscope (Nikon Eclipse Ci-L).

### Quantification of cells in p-ERK and NADPH-diaphorase staining

Sections of a set of wild-type and TNX-deficient spinal cords were concurrently immunostained, and images were captured under the same conditions. The p-ERK-positive or NADPH-diaphorase-positive cells were counted with NIH ImageJ software. The background intensity (arbitrary units) was measured in the white matter area of spinal cord. A cell was counted as positive cells if its intensity level was at least twice that of the background intensity level and if a single cell body was clearly defined. p-ERK immunoreactivity was quantified in 30 images (half of spinal cord) from 15 slices from the 3 separated wild-type or TNX^−/−^ mice. NADPH-diaphorase intensity was quantified in 27 (wild-type) or 32 (TNX^−/−^) images (half of spinal cord) from 16 slices prepared from the 4 separated wild-type or TNX^−/−^ mice. The numbers of respective positive cells were expressed as the mean ± S.E.M of the number in the indicated area (laminae I, II and III-V) of half spinal dorsal horn image.

### Statistical analysis

The data are expressed as the mean ± S.E.M. Statistical analysis was performed by the unpaired Student’s *t*-test (two-tailed) for comparisons between 2 groups. Group means were compared with a one-way ANOVA with a *post hoc* Bonferroni test and two-way ANOVA with a *post hoc* Bonferroni test. Differences were considered significant at *P* < 0.05. Statistical analyses were performed using the Excel Statistical Program ystat2006, created by S. Yamazaki (Igakutosho-shuppan, Tokyo, Japan), and Prism 6 software (GraphPad Software Inc., La Jolla, CA).

## Supplementary information

Supplementary information.
