# Supplementary material for: Mechanical allodynia in mice with tenascin-X deficiency associated with Ehlers-Danlos syndrome
Source: Sci Rep. 2020 Apr 16;10:6569. doi: 10.1038/s41598-020-63499-2 (PMC7162960; doi:10.1038/s41598-020-63499-2)
Supplement: Supplementary file 1 — Supplementary information. [file 41598_2020_63499_MOESM1_ESM.pdf]

Supplementary Information

Mechanical allodynia in mice with tenascin-X deficiency associated with Ehlers-Danlos syndrome

Emiko Okuda-Ashitaka[1,*], Yuka Kakuchi[1], Hiroaki Kakumoto[1], Shota Yamanishi[1], Hiroki Kamada[1], Takafumi Yoshidu[1], Satoshi Matsukawa[1], Naoya Ogura[1], Sadahito Uto[1], Toshiaki Minami[2], Seiji Ito[2], Ken-ichi Matsumoto[3]

[1]Department of Biomedical Engineering, Osaka Institute of Technology, Osaka 535-8585, Japan

[2]Department of Anesthesiology, Osaka Medical College, Takatsuki 569-8686, Japan

[3]Department of Biosignaling and Radioisotope Experiment, Interdisciplinary Center for Science Research, Organization for Research and Academic Information, Shimane University, Izumo 693-8501, Japan

*Corresponding author: e-mail address: emiko.ashitaka@oit.ac.jp

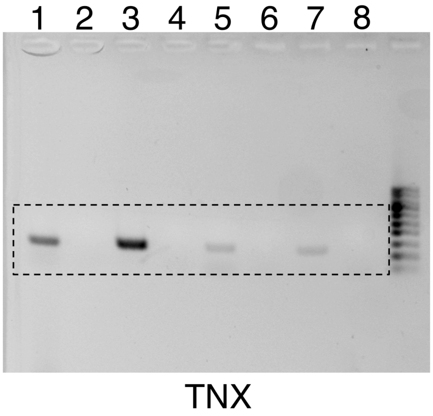

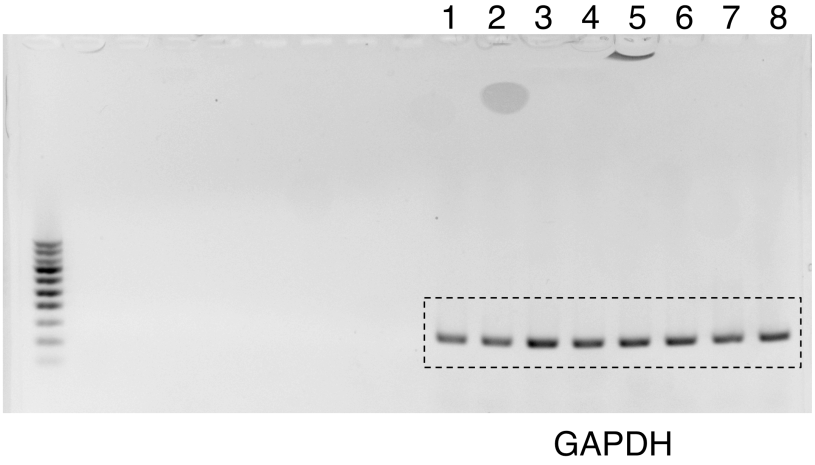

Supplementary Figure 1 Full-length gels in Figure 1a. The dotted rectangles indicate cropped parts of the gels.
